# The relationship between resilience and loneliness elucidated by a Danish version of the resilience scale for adults

**DOI:** 10.1186/s40359-020-00493-3

**Published:** 2020-12-10

**Authors:** Ida Skytte Jakobsen, Lykke Mie Riis Madsen, Martin Mau, Odin Hjemdal, Oddgeir Friborg

**Affiliations:** 1 Health, Social Work and Welfare Research, UCL University College, Odense, Denmark; 2 Department of Psychology, Norwegian University of Science and Technology, Trondheim, Norway; 3 Department of Psychology, UiT The Arctic University of Norway, Tromsø, Norway; 4 Department of Psychology, University of Southern Denmark, Odense, Denmark; 5 Health Sciences Research Centre, UCL University College, Odense, Denmark

**Keywords:** Loneliness, Resiliency, Protective factors

## Abstract

**Background:**

Research on the relationship between resilience and loneliness is sparse. The construct of resilience has been conceptualized in multiple ways, including the measurement of resilience. The Resilience Scale for Adults (RSA) is a measure of protective factors. The present study examined whether resiliency moderates any negative relationship between loneliness and mental health and additionally examined the psychometric properties of the Danish translation of the RSA.

**Methods:**

A Danish sample (*N* = 422) completed the UCLA Loneliness Scale, Hopkins Symptom Check List-25 (HSCL-25), the Sense of Coherence (SOC-13), and the RSA, Resilience Scale for Adults.

**Results:**

The measure of loneliness correlated significantly and negatively with most facets of the RSA, except the subscales of family cohesion and structured style. The strongest correlation was the negative one between loneliness and SOC. The results indicated that people feeling lonely also experience their life as less meaningful.

**Conclusion:**

The study supports the existing six-factor structure of the Resilience Scale for Adults (RSA) in a Danish sample. The results indicate that all facets of resiliency were negatively related to loneliness. Also, the facets of perception of self and family coherence could explain a substantial amount of the variance associated with symptoms of depression in relation to loneliness.

## Background

The social nature of human beings manifests itself as a basic need to belong [1], which, if not satisfied, may induce experiences of loneliness. To most people, loneliness is an aversive state of mind prompted by a discrepancy between the desired and actual quality of one’s interpersonal relationships [2]. Loneliness is subjective or individual, as objectively socially connected people may still feel lonely [3]. Numerous studies focusing on different age groups (for reviews see [1, 4, 5]) report an association between loneliness and different negative affective conditions. Among young adults, this raises awareness of loneliness as a risk factor for later negative affective problems [6], such as depression or anxiety [3], and eventually suicidal ideation as well as parasuicide [4]. Moreover, long-lasting loneliness may trigger additional problems such as stigmatization and social isolation [3] and may affect an individual’s cognition and behavior towards their social environment in a way that maintains feelings of isolation [7].

An interesting phenomenon is that not all chronically lonely people develop depression or anxiety, or develop other functional problems related to maladaptation. Some come to terms with their way of existence and may have adapted reasonably well. The present study examined this perspective by including variables that are protective of mental health.

Several concepts may be helpful in this regard. One is resilience as this research area delineates a range of factors that may promote adaptation and protect health despite exposure to adversity or hardships [8]. One may thus assume that well-adjusted lonely people may have more or better mental health protective factors available in their life. A relevant instrument in this regard is the Resilience Scale for Adults (RSA) [9], which the present study used.

The construct Sense of Coherence, as conceptualized by Antonovsky [10], is strongly related to positive mental health and adaptation in general and is a concept, which provides a cross-culturally validated comprehensive measure of positive mental health. According to Antonovsky [11], it represents” a generalized orientation toward the world which perceives it, on a continuum, as comprehensible, manageable and meaningful”. An individual with a high SOC has the capability to find meaning and purpose in life despite adversity. As such, it represents a general adaptation measure of stress. Moreover, high SOC persons have a positive expectation that new situations or challenges by conceiving them as comprehensible and manageable. The operationalization of the construct into the SOC-13 measure has been translated to more than 40 languages showing strong cross-culturally validity [12]. Given the considerably strong relationship with positive mental health as well as stress dampening effect of high SOC, we included the SOC-13 measure.

As a Danish version of the RSA is not yet tested or validated, the second aim of this study was to validate the original RSA. By added a measure of SOC to the study, we could learn both more about the association between SOC and RSA, and compare the RSA with a well-validated instrument.

### Resilience

The construct of resilience has been conceptualized in multiple ways, including the measurement of resilience [13]. Traditionally, resilience has been conceptualized as a certain *outcome*, e.g., ‘normal development under difficult conditions’ [14]. Such definitions may cause discussions about what constitutes ‘normal development’. Measurement of precursors of such end states may instead be more fruitful by providing knowledge about factors that promote favorable outcomes, which should be more valuable within a preventive or a clinical health perspective. The measurement of protective factors, as representative of this approach, may be preferable.

A review of existing self-rating resilience measures, [15] revealed no gold standard of measurement; yet, the RSA by [9] was positively rated. The RSA is a self-rating resilience measure that assesses protective factors across three broad classes: individual or personal, family, and social resources. These extra-individual factors as part of the RSA makes it well suited to examine loneliness as a social construct. The RSA was originally developed in Norway by [9]. Numerous later studies on Norwegian samples support its reliability and validity [16–22]. The cross-cultural validity is also accumulating, showing adequate support in for example in Peru [23], Pakistan [24], India [25, 26], Iran, [27], French-speaking Belgian [28], Brazil [29], in Lithuania [30] and in English with a sample from Australia [24]. An investigation of the psychometric properties of the RSA in a Danish sample has not yet been made, which was another impetus of the present study.

### Loneliness, resilience, and sense of coherence

Research on relationships between resilience and loneliness is so far sparse. Some loneliness studies on the elderly [31–34], young homeless people [35, 36], and on students [34], showing that resilience may protect and even mediate relationships between loneliness and health-related outcomes. We, therefore, expect similar favorable effects of adding the RSA to the current study of relationships between loneliness and mental health.

The Sense of coherence scale comprises three components that are combined in a single index: comprehensibility, manageability, and meaningfulness, which indicates a person’s global approach towards challenges, stressors, or adversities in their life. Antonovsky [10] argues that a person with a high SOC will more quickly analyze, understand, spot solutions, identify ways of appropriate coping, as well as finding meaning in dealing successfully with the implied challenges, which ultimately, improves that person’s general adaptability. It has been considered a part of protective factors that contribute to resilience and is negatively associated with loneliness [37, 38].

The present study examined the psychometric properties of the Danish translation of the RSA. The test score reliability was expected to be adequate. We also expected the RSA to correlate in expected directions variables related to loneliness and mental health (anxiety and depression), thus supporting convergent validity. Moreover, since the RSA is presumed to assess protective factors that in theory should dampen any negative health effects of risk variables, such as loneliness, we additionally expected the RSA to moderate (or dampen) any negative relationship between loneliness and mental health.

## Method

### Subjects

Participants were recruited from the UCL University College in Odense, Denmark. Data were collected via an online survey, which was distributed to 575 university students. The response rate was 73.4% as 422 students completed the survey (136 male-32%, and 284 female – 68%). The sample consisted of first-year students studying pedagogics (36%), nursing (16%), biomedical laboratory science (12%), teaching (3%), or the full-degree business academy program (33%). Their age ranged from 19 to 56 years (*M* = 25.4, *SD* = 6.18) with females 25.4 years in average (*SD* = 6.48) and males being 25.6 years (*SD* = 5.57).

### Procedures

The initial contact was to the headmaster at each department, explaining the aim of the study and the procedure. Hereafter, the students could access the questionnaire through their online student platform. All students were explained the purpose and confidentiality of the study, and that participation was voluntary. Data was collected from August 2018 to December 2018.

### Demographics

Information about the students’ gender, age, and marital status were gathered. Race, ethnicity, and income were not covered.

## Instruments

### UCLA loneliness scale

The Three-Item Loneliness Scale (T-ILS) is a short version adapted from the standard measure of loneliness, the Revised UCLA Loneliness Scale. The short version of the scale has demonstrated concurrent validity and good internal consistency [39]. The T-ILS includes three items: “1) How often do you feel left out?”, “2) How often do you feel that you lack companionship?”, and “3) How often do you feel isolated from others?” Responses are scored on a three-point scale ranging between 1-“hardly ever”, 2-“some of the time” and 3-“often”. A principal component analysis of this scale in the present study yielded an eigenvalue of 2.27 (*R*^2^ = 76%), clearly supporting the adequacy of creating a general loneliness index as the average of these three items (higher scores indicating more loneliness).

### Resilience scale for adults (RSA)

The RSA [9] is a 33 item self-report scale developed for measuring protective resilience factors among adults. It assesses an individual’s available resilience resources across an intrapersonal domain (perception of self, perception of future, social competence and having a structured style), and across a focal interpersonal domain (i.e., cohesion in the family) and a more distal interpersonal domain (i.e., extra-family social resources). The instrument uses a seven-point semantic differential scale in which each item has a positive and a negative attribute at each end of the scale [17]. Half of the items are reversely scored to reduce acquiescence-biases. In the questionnaire, the items constructing the five subscales are mixed. An example of an item is ‘My family is characterized by’: where one end of the scale goes from Healthy cohesion to Disconnection.

Higher scores indicate higher levels of protective resilience factors with an adequate measurement reliability (alpha ranging between ~.70 and ~ .85 [40, 41];. The construct and cross-cultural validity of the RSA is well documented, and it is a recommended resilience scale [15].

### The Hopkins symptom check List-25 (HSCL-25)

We used a Danish version of the instrument HSCL-25 [42], a self-report instrument that measures psychopathological symptoms (i.e. depression, anxiety, and total distress). It contains 13 depression items, 10 anxiety items, and 2 somatic items. All items have a Likert scale with four categories (“Not at all,” “A little,” “Quite a bit,” “Extremely,”), where higher scores indicate higher levels of psychiatric or affective symptoms. HSCL-25 is one of the most widely used screening instruments for psychopathologic symptoms [43] with reports of good internal consistency (standardized Cronbach’s *α*) generally, *α* > .90 for the total score, and *α* > .80 for anxiety and depression [44]. It has been found to be a valid screening instrument in both Western and non-Western populations [45–47] with some exceptions [48].

### Sense of coherence (SOC-13)

The SOC-13 is a brief version of the SOC-29 self-report questionnaire [49]. The instrument measures Sense of Coherence, which has been associated with resilience, thus indicating that it is a factor in determining one’s ability to cope with harsh events [50]. In addition, the instrument has in former studies demonstrate significant positive correlations with RSA [16].

SOC-13 measures psychological constructs that comprise Sense of Coherence, namely: comprehensibility (cognitive), manageability (instrumental/behavioral), and meaningfulness (motivational) [51]. We have used the adapted SOC-13, which has been translated into Danish. The scale is introduced, as “Here is a series of questions relating to various aspects of our lives”. Each question has five possible answers [52]. Higher scores indicate higher levels of SOC and thus a higher level of individual adjustment.

The Sense of Coherence (SOC-13) a reliable instrument for measuring the individual’s potential adjustment and rehabilitation to stressful life experiences [51, 53]. Exemplar items are: 1) “Do you have the feeling that you are in an unfamiliar situation and don’t know what to do? (Comprehension), 2) “How often do you have feelings that you’re not sure you can keep under control?” (Manageability), and 3)“How often do you have the feeling that there’s little meaning in the things you do in your daily life?” (Meaningfulness).

### Statistics

SPSS 25 and Mplus 7.4 [54] was used for all inferential and confirmatory factor analyses (CFA), respectively.

In Mplus, the robust ML (maximum likelihood) estimator was used to adequately adjust for non-normal item score distributions. As the chi-square absolute fit measure is sensitive to large sample sizes [55], the root-mean-square error of approximation (RMSEA) and the non-normed fit index (NNFI) were additionally consulted. RMSEA values < .06 are preferable [56], while values for NNFI should minimally pass > .90 [57] or preferably > .95 [58]. Standardized root mean residuals should ideally be less than < .08, which represents the average size of the residual item correlations after fitting the factor model.

The regression analyses were bootstrapped using 1000 resamplings in order to produce confidence intervals and significance tests less biased by non-normally distributed scores, as was the case for the HSCL depression and anxiety scores.

Beta coefficients with *p-*value < .05 was deemed as statistically significant. The regression models were conducted in steps. All continuous variables were grand centered, and dichotomous variables were dummy coded (0 versus 1). In the first step, we entered loneliness, thus yielding its crude or unadjusted relationship with anxiety or depressive symptoms. In the second and third step, the resilience variables and the SOC measure was added, respectively. In the final block, their interaction terms were additionally included (loneliness × RSA or loneliness × SOC) along with a final adjustment by including the covariates (e.g., age, gender, and education). The performance of these models was gauged with the adjusted R-square index (range 0–1) indicating the degree of variance explained by the model.

## Results

### Descriptive statistics

The score range, means (or proportions), standard deviations of all variables, as well as the reliability coefficients of the measurement scales, are presented in Table 1. The interrelationships between these variables are given as Pearson correlation coefficients. The psychometric properties of the RSA, the HSCL-25, and SOC-13 were adequate.

**Table 1:** Pearson correlations coefficients, Cronbach’s alphas and descriptive statistics for the measurement variables (*N* = 422)

	Variables														
	1	2	3	4	5	6	7	8	9	10	11	12	13	14	15
*Outcome*															
1 HSCL anx	**.86**														
2 HSCL depr	.66	**.90**													
*Risk variable*															
3 Loneliness	.46	.62	**.84**												
*Protection*															
4 RSA pc	−.58	−.66	−.51	**.85**											
5 RSA fut	−.49	−.67	−.51	.68	**.88**										
6 RSA sc	−.34	−.50	−.56	.49	.51	**.81**									
7 RSA fc	−.34	−.46	−.38	.35	.42	.43	**.87**								
8 RSA sr	−.41	−.57	−.57	.48	.54	.56	.68	**.87**							
9 RSA ss	−.15	−.35	−.20	.30	.45	.19	.31	.29	**.66**						
10 SOC	−.63	−.77	−.67	.72	.68	.52	.49	.61	.40	**.90**					
*Covariates*															
11 Age	−.10	−.04	−.04	.23	.09	.04	−.07	−.03	.12	.10					
12 Marital stat.	.12	.22	.19	−.20	−.18	−.09	−.13	−.15	−.22	−.24	−.27				
13 Children	.08	.11	.07	−.24	−.17	−.09	−.02	−.03	−.19	−.18	−.69	.39			
14 Pets	−.03	−.04	−.11	.02	.05	.06	.03	.06	−.05	.05	−.19	.15	.18		
15 Sports	.05	.14	.17	−.12	−.18	−.24	−.14	−.13	−.23	−.16	−.03	.00	.00	−.09	
*Descriptive data*															
Range	1–4	1–4	1–3	1–7	1–7	1–7	1–7	1–7	1–7	1–5	19–56	0–1	0–1	0–1	0–1
*M* or %	1.51	1.52	1.65	4.56	5.18	4.85	5.25	5.89	4.71	3.40	25.44	.61	.18	.29	.64
*SD*	.47	.51	.68	1.32	1.39	1.23	1.28	1.03	1.26	0.66	6.18	.49	.39	.46	.48

Correlation coefficients above xxx and xxx are significant at the 0.05 and 0.01. Cronbach’s Alpha of the measurement scales are presented in the diagonal as bold text. RSA pc/fut/sc/fc/sr/ss = personal competence / planned future / social competence / family cohesion / social resources / structured style

The reliability coefficients for the subscales of the RSA were acceptable as the Cronbach’s alphas varied in the range between .81–.87 for the subscales planned future, family cohesion, social resources, personal competence, and social competence (in falling order). The subscale “structured style” was however in the sub-optimal range (α = .66).

Loneliness was in general strongly associated with higher levels of anxiety and depressive symptoms. Moreover, loneliness correlated significantly and negatively with most facets of the RSA, except for the subscales of family cohesion and structured style. The strongest correlation was the negative one between loneliness and SOC, thus indicating that people feeling lonely also experience their life as less meaningful, comprehensible, and manageable. Moreover, the RSA and the SOC were strongly positively correlated, as has been previously reported [19].

### Confirmatory factor analysis

The fit of the six-factor RSA measurement model was examined in a confirmatory factor analysis, which confirmed adequate fit in terms of a low degree of model misspecification (RMSEA = .052, *CI*_.95_ .048–.056; SRMR = .066), whereas the relative fit was mediocre (CFI = .898, TLI = .888). The modification indices indicated that one item originally belonging to the social resource factor (*becoming informed if a family member experiences a crisis*) loaded strongly on the family cohesion factor (λ = .65). Switching this item to the family cohesion factor, which is reasonable given the overlap in semantic meaning, improved absolute fit (RMSEA = .049, *CI*_.95_ .045–.054, SRMR = .063) and relative fit (CFI = .909, TLI = .900). The standardized factor loadings are given in Table 2.

**Table 2:** Factor loadings of the resilience scale for adults following a confirmatory factor analysis (*N* = 422)

*Items*	Personal competence	Planned future	Social competence	Family cohesion	Social resources	Structured style
*Factor 1*						
RSA1	.70					
RSA7	.67					
RSA13	.69					
RSA19	.77					
RSA25	.77					
RSA29	.59					
*Factor 2*						
RSA2		.78				
RSA8		.83				
RSA14		.81				
RSA20		.80				
*Factor 3*						
RSA3			.46			
RSA9			.56			
RSA15			.83			
RSA21			.86			
RSA26			.45			
RSA30			.69			
*Factor 4*						
RSA4				.61		
RSA10				.83		
RSA16				.75		
RSA22				.77		
RSA27				.73		
RSA31				.67		
*Factor 5*						
RSA5					.70	
RSA11					.78	
RSA17					.64	
RSA23				**.63**		
RSA28					.83	
RSA32					.69	
RSA33					.79	
*Factor 6*						
RSA6						.44
RSA12						.32
RSA18						.75
RSA24						.74

### The relationship between loneliness and mental health, and the contributing role of RSA and SOC as protective factors (or moderators)

Loneliness was regressed upon depression (Table 3) and anxiety (Table 4) and stratified to retain gender-specific effects. As a single variable (crude effect), loneliness had the highest association with depressive symptoms in men (*R*^*2*^ = 41%), thereafter depressive symptoms in women (*R*^*2*^ = 38%), and then anxiety in both men and women (*R*^*2*^ = 20 and 21%, respectively). Adding resilience to the equation in the second block explained substantially more of the variance in mental health, thus validating the Danish version of the RSA as a significant contributor in explaining mental health. Adding SOC in the third block, explained a substantial extra amount of the variance in the HSCL, as expected.

**Table 3:** Multiple regression analyses with HSCL depression as the dependent variable (*N* = 420)

		Men (*n* = 136)						Women (*n* = 284)				
	*R-sq*	*Crude*	*Adj 1*	*Adj 2*	*Adj 3*	*CI 95%*	*R-sq*	*Crude*	*Adj 1*	*Adj 2*	*Adj 3*	*CI 95%*
*Risk variable*	.406						.384					
Loneliness		.75^a^	.36^a^	.14	.13	−.08 | .32		.57^a^	.23^a^	.15^b^	.11^c^	.00 | .21
*Protection*	.600						.617					
RSA pc			−.26^b^	−.12	−.17^c^	−.35 | .01			−.29^a^	−.16^a^	−.19^a^	−.29 | -.09
RSA fut			−.23^b^	-.16^c^	−.14	−.30 | .05			−.24^a^	−.20^b^	−.17^b^	−.29 | -.03
RSA sc			.10	.02	−.01	−.19 | .18			−.03	−.01	.00	−.09 | .08
RSA fc			−.19^c^	−.08	−.18^c^	−.36 | -.02			−.06	−.05	−.06	−.19 | .06
RSA sr			−.10	−.11	.06	−.18 | .32			−.06	.00	.04	−.12 | .18
RSA ss			−.04	.03	.04	−.09 | .19			−.03	.00	−.03	−.12 | .06
SOC	.661			−.47^a^	-.38^b^	−.59 | -.17	.649			−.34^a^	−.32^a^	−.44 | −.20
*Interactions*	.712						.678					
Lonely × RSApc					−.16^c^	−.30 | .02						
Lonely × RSAfc					−.20^b^	−.34 | −.06						
Lonely × SOC											−.17^a^	−.25 | −.09
*Covariates*	.706						.685					

^a^ *p* < .001, ^b^ *p* < .01 and ^c^ *p* < .05. No. of resamplings = 1000. Covariates were: Age (yrs), marital status (single/cohabitation), children (no/yes), Education (teacher, nurse, biomechanics vs other), pets (no/yes), engage in sports (no/yes). RSA pc/fut/sc/fc/sr/ss = personal competence / planned future / social competence / family cohesion / social resources / structured style, SOC = Sense of Coherence

**Table 4:** Multiple regression analyses with HSCL anxiety as the dependent variable (*N* = 420)

		Men (*n* = 136)						Women (*n* = 284)				
	*R-sq*	*Crude*	*Adj 1*	*Adj 2*	*Adj 3*	*CI 95%*	*R-sq*	*Crude*	*Adj 1*	*Adj 2*	*Adj 3*	*CI 95%*
*Risk variable*	.195						.209					
Loneliness		.43^a^	.16	−.08	−.05	−.24 | .11		.46^a^	.18^c^	.09	.09	−.05 | .23
*Protection*	.382						.386					
RSA pc			−.28^a^	−.14	−.16^c^	−.31 | −.01			−.43^a^	−.29^a^	−.27^a^	−.42 | −.11
RSA fut			−.10	−.02	.01	−.13 | .15			−.14	−.10	−.12	−.32 | .08
RSA sc			.12	.04	.04	−.1 | .18			.06	.08	.07	−.08 | .22
RSA fc			−.19^b^	−.08	−.13	−.26 | .00			−.06	−.04	−.05	−.19 | .10
RSA sr			−.04	−.04	.06	−.16 | .28			−.08	−.03	−.02	−.22 | .17
RSA ss			−.07	.00	−.02	−.12 | .08			.15^c^	.19^c^	.17^c^	.04 | .31
SOC	.486			−.49^a^	−.49^a^	−.69 | −.30	.416			−.37^a^	−.38^a^	−.57 | −.18
*Interactions*	.542											
Lonely × RSApc					−.22^b^	−.37 | −.02					ns	
Lonely × SOC					ns						ns	
*Covariates*	.519						.414					

^a^ *p* < .001, ^b^ *p* < .01 and ^c^ *p* < .05. No. of resamplings = 1000. Covariates were: Age (yrs), marital status (single/cohabitation), children (no/yes), Education (teacher, nurse, biomechanics vs other), pets (no/yes), engage in sports (no/yes). RSA pc/fut/sc/fc/sr/ss = personal competence / planned future / social competence / family cohesion / social resources / structured style, SOC = Sense of Coherence

In the final block, RSA and SOC were added as moderators of the relationship between loneliness and HSCL in order to examine if these two respective was associated with an extra layer of protection in addition to their compensatory main effects. The RSA contributed significantly as moderators of depressive symptoms in men (notably, the subscales of perception of self and family coherence) and anxiety symptoms in men (RSA perception of self). Similar protective effects against depressive symptoms were not observed in women, whereas SOC was associated with a protective role against depressive symptoms in women. These findings indicated that both RSA and SOC showed compensatory (main) and protective (moderator) effects. The latter effects were more pronounced for the RSA measure.

## Discussion

The current study showed that loneliness was related to both anxiety and depression and that all facets of resiliency were negatively related to loneliness, where higher loneliness was associated with lower resiliency, indicating that young adults who show a high degree of resilience also tended to feel less lonely. The relation between levels of loneliness and resilience is particularly interesting as it has not previously been reported, but it also supports the construct validity of the RSA.

A possible explanation for the relation between levels of resilience and levels of loneliness is that resilience represents the presence of both intra- and interpersonal resources that improve the adaptation to a more lonely existence. Similarly, situational characteristics, such as having few social resources, shallow or non-existing interpersonal relationships, are hypothesized as a predisposing factor for developing loneliness [59].

Thus, higher scores on loneliness, indicating the absence of social resources, would thus be expected to be associated with lower scores related to social resources such as resilience. Social resources are thought to be essential in mental health as researchers highlight that healthy adaptation is a process [8, 15, 60]. More specifically, it can be defined as a *transactional process* where resilience is developed through the individuals’ dynamic interaction with their environment. It may also be described as the individual’s ability to navigate between available resources [61].

Based on the narrow definition and measurement of loneliness in the present study, we cannot ascertain to what extent lonely people have or use social resources despite observing a strong negative correlation between loneliness and social resources. According to the model of loneliness on cognition [7], feelings of loneliness are maintained through the individual’s interaction with his or her social environment. Feelings of loneliness change cognitive expectations that may reinforce maladaptive behavior, e.g., hesitance, submissiveness, or withdrawal related to perceptions of the social sphere as threatening that also shapes memories of social interactions as more negative as compared to non-lonely people [7].

Loneliness as a subjective experience is typically distinguished from e.g. social isolation, which describes social circumstances more objectively. Future studies into the association between loneliness and resiliency could therefore benefit from a multi-dimensional approach to the study of loneliness encompassing both subjective and objective dimensions [62].

Furthermore, this study illustrated, that loneliness was strongly associated with worse mental health, and in particular, depression. This association between loneliness and other mental health problems adds to an understanding of loneliness as a complex phenomenon [6] associated with a range of challenges. This finding has also been replicated elsewhere in the literature, where loneliness seems to correlate with other mental health problems in reciprocal relationships [3, 63, 64].

However, this study showed that resilience, specifically the facets of perception of self and family coherence, could explain a substantial amount of the variance associated with symptoms of depression in relation to loneliness, and the facet of perception of self was associated with anxiety in relation to loneliness.

The measure of RSA and the SOC are positively correlated which indicates that they both measure individual resources. The design of the study makes it impossible to detect any causal relation between the two or a causal relation between loneliness and SOC. The study can only report on a negative correlation between SOC and UCLA, which we can discuss from a theoretical perspective. The negative correlation is indicating that people feeling lonely also experience their life as less meaningful, comprehensible, and manageable. The social nature of humans makes relationships and the sense of belongingness a core component of how creates meaning in our lives. A key channel for humans to make lives understandable and comprehensible is to discuss, engage, and interact with other humans. In his original work from 1979, Antonovsky [65] argued that life experiences shape the sense of coherence and that SOC is a stable entity around the age of 30. Since then a number of studies have shown that interventions can influence SOC levels and interventions focusing on strengthening SOC make be an important element in the effort to combat loneliness [66]..

The psychometric properties of the Resilience Scale for Adults (RSA) were supported with support for the existing six-factor structure and good reliability in a Danish sample, which indicates that this Danish version may be interesting when exploring levels of resilience. Further, the study explored the construct validity of the RSA. Since this is the first study reporting on the validity of the RSA in a Danish population, it is noteworthy that the overall results (Table 1) indicate that the construct validity of the RSA is supported. The significant positive correlations with Sense of Coherence, which measures adaptation in general, supports the construct validity of the RSA. As previously reported [9, 19] the magnitude of the correlation between the SOC and the individual RSA factors vary, indicating that different RSA factors relate differently to SOC and do not overlap. The construct validity is further supported by the significant negative correlations with the Hopkins Symptom Check List and thus levels of anxiety and depressive symptoms.

### Limitations

One limitation of the present study is the young age of the participants. In addition, all participants were university students and the majority were young women. This implies caution concerning generalization, as this sample is not representative of the general adult population in Denmark. Further validity studies of the RSA on more heterogeneous samples in terms of age and occupation may address this uncertainty. However, given the ever-growing base of studies confirming the validity of the RSA, we except it to generalize beyond this university sample.

A second limitation is that the study applied a cross-sectional design, and therefore our hypotheses direction and causality between loneliness and possible protective factors needs to be examined in future studies. Especially studies using longitudinal designs with repeated measures that would allow more causally related inferences as well as the identification of various trajectories of lonely people related to their mental as well as somatic health.

Age was adjusted for in the current study and as the adjusted statistical effect of loneliness became rather small or non-significant after adding the other covariates, and in particular sense of coherence, any further nuanced analyses of age should be of minor importance. However, given that, loneliness is manifested differently among young and older adults, further studies are called for, in order to shed some light upon a more comprehensive understanding of the implication of age.

## Conclusion

The current study showed that loneliness measured by the UCLA was negatively related to all facets of resiliency measured by the RSA. The psychometric properties of the RSA were supported in a Danish sample reproducing the original factor structure. As specific aspects of the RSA could explain a substantial part of the variance in anxiety and depressive symptoms associated with loneliness, resiliency may be an important concept to consider in the loneliness research. The need for additional research is especially evident in relation to interventions targeted loneliness among young people, as interventions aimed at reducing loneliness levels have showed only negligible effects [6].

As the concept of resiliency can inspire interventions targeted prevention or reduction of mental health problems [58], we encourage future studies to examine more closely how resiliency factors and resilience may moderate the association between mental health problems and loneliness.

## Data Availability

The datasets used and analyzed during the current study are available from the corresponding author on reasonable request.

## Abbreviations

CFI: Comparative fit index
HSCL-25: The Hopkins Symptom Check List-25
ML: Maximum likelihood
NNFI: Non-normed fit index
RMSEA: Root-mean-square error of approximation
RSA: The Resilience Scale for Adults
SOC-13: The Sense of Coherence Scale
SRMR: Standardized root mean square residual
T-ILS: The Three-Item Loneliness Scale
TLI: Tucker-Lewis index
